# Evaluation of the Effect of Hypercapnia on Vascular Function in Normal Tension Glaucoma

**DOI:** 10.1155/2015/418159

**Published:** 2015-10-18

**Authors:** B. Quill, E. Henry, E. Simon, C. J. O'Brien

**Affiliations:** ^1^UCD School of Medicine and Medical Science, University College Dublin, Belfield, Dublin, Ireland; ^2^Princess Alexandra Eye Pavilion, Edinburgh, UK

## Abstract

*Introduction*. Altered ocular perfusion and vascular dysregulation have been reported in glaucoma. The aim of this paper was to evaluate the vascular response to a hypercapnic stimulus. *Methods*. Twenty normal tension glaucoma (NTG) patients and eighteen age- and gender-matched controls had pulsatile ocular blood flow (POBF) measurements, systemic cardiovascular assessment, and laser Doppler digital blood flow (DBF) assessed. Measurements were taken at baseline, after 10-minutes rest, in the stable sitting and supine positions and following induction and stabilization of hypercapnia, which induced a 15% increase in end-tidal pCO_2_. The POBF response to hypercapnia was divided into high (>20%) and low responders (<20%). *Results*. 65% of NTG patients had a greater than 41% increase in POBF following CO_2_ rebreathing (high responders). These high responders had a lower baseline POBF, lower baseline DBF, and a greater DBF response to thermal stimulus. *Conclusion*. NTG patients that have a greater than 20% increase in POBF after a hypercapnic stimulus have lower baseline POBF and DBF values. This suggests that there is impaired regulation of blood flow in a significant subgroup of NTG patients. This observation may reflect a generalised dysfunction of the vascular endothelium.

## 1. Introduction

Altered blood flow mechanisms are thought to play a role in glaucoma. Multiple studies have shown that a reduction in perfusion pressure [1–7], vasospastic function [8–14], and a disturbance in autoregulation [10, 11, 15–18] are involved in the pathogenic processes. The past thirty years has seen a renewed interest in vascular endothelial function in ocular and systemic blood flow regulation [19–24]. Blood flow abnormalities in glaucoma have been documented by using different techniques, such as fluorescein angiography [25], colour Doppler imaging [26], laser Doppler flowmetry [27], and pulsatile ocular blood flow (POBF) measurements [28]. Glaucomatous patients with low systolic perfusion pressure at baseline have been shown to have a considerably faster progression in visual field loss [29].

The vascular endothelium plays a key role in angiogenesis, inflammatory responses, haemostasis, and control of vascular tone [30]. In vitro and in vivo studies have provided evidence for the role of the endothelium in the control of blood flow in the retina, optic nerve head (ONH), and choroid. Impaired endothelial vascular function has been demonstrated in normal tension glaucoma (NTG) and other glaucomas [3, 21, 31–33]. These studies indicate that endothelial dysfunction in NTG patients is not confined to the ocular vasculature but can be seen in the systemic circulation too. The vascular endothelium normally maintains vascular tone through autocrine, paracrine, and endocrine-like functions [24]. Abnormalities of the l-arginine/nitric oxide (NO) and endothelin-1 (ET-1) systems have been implicated in the pathophysiology of NTG [32, 34]. The possibility that ET-1 contributes to vasospasm in ocular diseases such as NTG is supported by the demonstration of elevated basal plasma ET-1 concentrations in patients [35, 36], combined with an abnormal response of plasma ET-1 concentrations to postural [37] and temperature changes [38]. The association of ET-1 involvement in NTG is further enhanced by the animal model of ONH ischaemia with repeated perineural injections of ET-1 [39].

Carbon dioxide (CO_2_) is an extremely potent cerebrovascular and ocular vasodilator [40, 41]. Harris et al. proposed that vasospasm distal to the ophthalmic artery may be present in patients with normal tension glaucoma and can be reversed with the administration of a cerebral vasodilator [42]. It has been demonstrated that, in normal control subjects, breathing CO_2_ causes vasodilation and thereby leads to an increase of retinal and ONH blood flow [43–49]. There is evidence that some NTG patients show a marked improvement in visual field when exposed to CO_2_ due to its vasodilatory properties [50]. In NTG an exaggerated response of ocular blood flow to hypercapnia has been demonstrated previously [51].

Pulsatile ocular blood flow (POBF) measures only the pulsatile component of ocular blood flow which accounts for about 80% of total flow and is predominantly derived from the choroidal circulation [52]. The posterior ciliary arteries are the main arterial supply to the choroid and also represent the main arterial vascular supply to the anterior optic nerve [53]. POBF is generally reduced in untreated primary open angle glaucoma (POAG) [54]. IOP also affects POBF. Pharmacologically lowering IOP results in a significant increase in mean POBF [55].

The aim of this study was to initially evaluate vascular response to a hypercapnic stimulus at an ocular and systemic level. Following on from this we were keen to explore the reason for the differential responses to the vasodilatory stimulus.

## 2. Methods

### 2.1. Subject Selection

Thirty-eight patients (20 NTG and 18 controls) were recruited prospectively over a 28-month period from glaucoma clinics at the Princess Alexandra Eye Pavilion, Edinburgh. An experienced glaucoma specialist confirmed the clinical diagnosis of NTG in all cases. All but four of the patients were newly diagnosed with NTG and were enrolled to and participated in the study prior to commencing any antiglaucoma medication. The four patients who had previously been on treatment underwent a washout period prior to participation.

All subjects were Caucasian and were matched for age, sex, and axial and forearm length. The Lothian Research Ethics Committee granted ethical approval for the study and the tenets of the Declaration of Helsinki were observed in all aspects of the study. Written, informed consent was obtained from each subject prior to enrolment in the study, following detailed explanation of the study procedures.

### 2.2. Investigations

A full ocular and systemic history was then taken. Subjects were questioned with regard to cardiovascular disease and history of past hypovolemic or hypotensive events. Subjects were also assessed for migraine using a questionnaire based on the International Headache Society guidelines. Likewise the presence of Raynaud's type peripheral circulation was assessed using well recognized criteria (Figure 8) [56–59]. Intraocular pressure was measured with a Goldmann tonometer and was recorded at two hourly intervals throughout the day. Visual field examination was performed using automated perimetry on the Humphrey perimeter (Humphreys Instruments, Inc., Allergan Humphrey, San Leandro, CA, USA) using the 24-2 threshold programme.

### 2.3. Measurement of Pulsatile Ocular Blood Flow

Ocular blood flow measurements were measured using the system devised by OBF Labs (System 3000, Version 14.4, Revision 4, OBF Labs, UK) based on the system devised by Langham and colleagues [60].

Subjects were connected to a respirometer linked to a DATAX carbon dioxide (CO_2_) analyser measuring end-tidal carbon dioxide, reflecting arterial pCO_2_. Systemic blood pressure was recorded at each stage of the measurements using an automated sphygmomanometer (Critikon, Dinamap, Takeda, Florida). Heart rate was recorded by a pulse oximeter. In patients with bilateral NTG, the eye with the greater degree of optic disc cupping and visual field loss was assessed. In all control subjects the right eye was assessed.

Ocular blood flow measurements were taken using the system devised by OBF Labs (System 3000, Version 14.4, Revision 4, OBF Labs, UK). Measurements were taken at baseline, after 10-minute rest, in the stable sitting and supine positions and following induction and stabilization of hypercapnia with inhalation of a mixture of 95% oxygen and 5% carbon dioxide (Carbogen), which induced a 15% increase in end-tidal pCO_2_.

IOP, pulse amplitude, pulse volume, systemic blood pressure, and heart rate were recorded in all subjects for each of the three measurement situations. Mean arterial blood pressure was calculated according to the equation:

(1) $$\begin{array}{c} \text{MAP}=\text{Diastolic BP}+\frac{1}{3}\left(\text{Systolic BP}-\text{Diastolic BP}\right). \end{array}$$

Changes in all of the parameters, HR, MAP, IOP, PA, PV, and POBF, from sitting to supine and following induction of hypercapnia were calculated for each individual subject and mean group responses were then calculated.

### 2.4. Digital Blood Flow

In this study digital blood flow (DBF) was measured using the periflux Pfz Laser Doppler flowmeter (Perimed KB, PO Box 5607, Stockholm, Sweden) with a 2 mW-NE laser of wavelength 632.8 nm. After a fifteen-minute rest period, the laser Doppler flowmeter (LDF) probe was placed lightly on the pulp of the middle finger and baseline blood flow was recorded in real time. This hand was then immersed in warm water (40°C) for 2 minutes and then wrapped in a towel to maintain the warmed temperature. Finger blood flow was again recorded until it reduced to baseline. The hand was then immersed in iced water (4°C) for 20 seconds and blood flow was recorded continuously until it returned to baseline levels.

Baseline, maximum and minimum blood flow and recovery to baseline times were all calculated. Ratios of maximum flow: baseline and baseline: minimum flow were deduced for each subject.

### 2.5. Statistical Analysis

A one-way analysis of variance was used to compare the demographics of the two groups and their systemic haemodynamic parameters and ocular blood flow characteristics. *t*-testing was used for intragroup comparisons to assess the effects of postural change and hypercapnia on these parameters. Intergroup comparisons were made of the mean flow ratios and recovery times using ANOVA.

## 3. Results

### 3.1. Group Demographics

Comparison of demographic data (Table 1) demonstrated that glaucoma patients were well matched with controls: there were no significant differences in age, sex, intraocular pressure (Goldmann), or axial length between the two groups. Baseline systemic haemodynamic parameters, namely, mean arterial blood pressure (MAP) and heart rate (HR), were also similar in the two groups. Eight patients and 3 controls had a history of borderline hypertension; none had received any antihypertensive medication and all were under observation by their general practitioners. Significant differences existed between the 2 groups for other medical history. Five patients gave a past history of a hypovolemic or profound hypotensive episode compared with none of the controls (*P* = 0.018). 11 patients gave a history of migraine and/or Raynaud's type peripheral circulation compared with 4 controls (*P* = 0.008) (Table 1).

### 3.2. Intraocular Pressure

Intraocular pressure was slightly higher in the NTG group throughout measurements but this was not significant. IOP increased slightly in both groups in the supine position but did not reach significance (NTG 3.8%; control 3.6%). Hypercapnia had minimal effect on IOP with a very small drop of less than 0.5 mmHg found in both groups.

### 3.3. Systemic Haemodynamic Parameters

**Figure 1:**
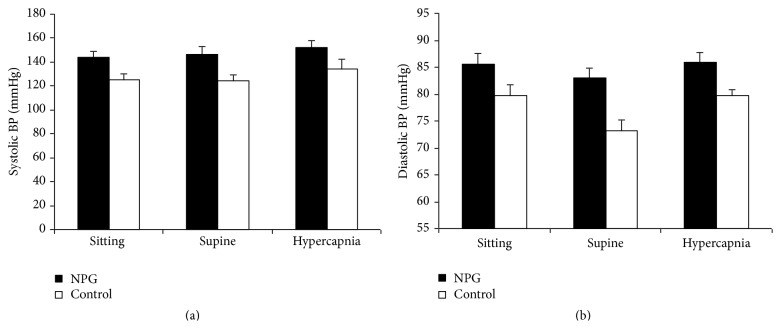
Blood pressure during pulsatile ocular blood flow studies. Systolic and diastolic blood pressure (BP) in millimeters of Mercury (mmHg) in normal tension glaucoma patients (NTG) and healthy volunteers (control) in sitting and supine positions and following induction of hypercapnia. Results are expressed as mean ± standard error of mean.

Baseline mean arterial blood pressure (MAP) and heart rate (HR) were similar in the two groups. Systolic blood pressure (SBP) increased slightly in both groups in the supine position (NTG: 1.53%; control: 0.6%). Both groups experienced an increase with hypercapnia, a known physiological response to hypercapnic-induced release of catecholamines, but this was only significant in the control group (NTG: 3.78%, *P* = 0.09; controls: 8.36%, *P* = 0.041) (Figure 1(a)).

Diastolic BP was higher in the glaucoma group in all three situations. This was only significant in the supine position. Diastolic BP fell in the supine position by 2.69% in NTG (*P* = 0.1) and 7.5% in controls (*P* = 0.04). With hypercapnia there was an increase in both groups with only the control group showing significance (NTG: 3.46%, *P* = 0.1; controls: 18.87%, *P* = 0.028) (Figure 1(b)).

**Figure 2:**
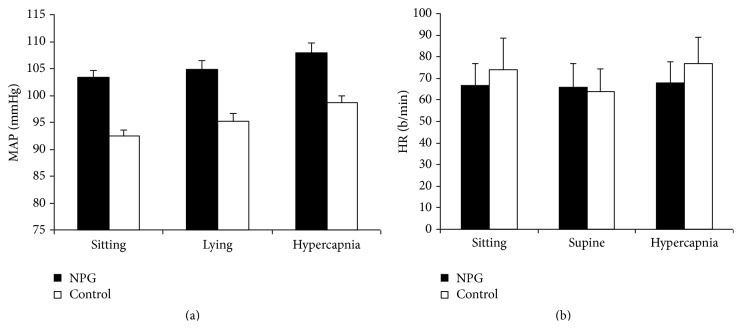
Blood pressure and heart rate during pulsatile ocular blood flow studies. Mean arterial blood pressure (MAP) in millimeters of Mercury (mmHg) and heart rate (HR) in beats per minute (b/min) in normal tension glaucoma patients (NTG) and healthy volunteers (controls) in sitting and supine positions and on induction of hypercapnia. Results are expressed as mean ± standard error of mean.

Mean arterial blood pressure was higher in the glaucoma group in all three situations but never achieved significance (Figure 2(a)). Supination caused a minimal increase in MAP in both groups (NTG: 1.4%; control: 2.8%). Hypercapnia induced a mean 3.28% increase in NTG that was not significant but the control group showed a mean increase of 20.3% (*P* = 0.03).

Heart rate tended to be higher in the control group in sitting (*P* = 0.09) and hypercapnic conditions (*P* = 0.04) (Figure 2(b)). Control patients demonstrated a significant drop in HR on lying down (16.88%  *P* = 0.045) and an increase with hypercapnia (*P* = 0.031). This was not observed in NTG patients who showed a 2.24% decrease in the supine position (*P* = 0.2) and an increase of 3.28% with hypercapnia (*P* = 0.1).

### 3.4. Ocular Blood Flow Characteristics

#### 3.4.1. Pulse Amplitude

**Figure 3:**
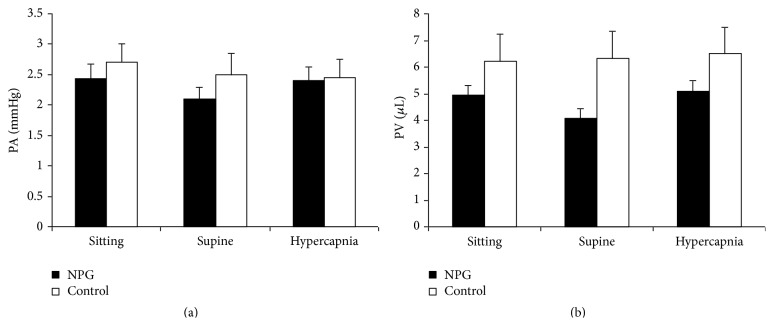
Pulse amplitude and pulse volume. Pulse amplitude (PA) in millimeters of Mercury (mmHg) and pulse volume (PV) in microlitres (*μ*L) in normal tension glaucoma patients (NTG) and healthy volunteers (controls) in sitting and supine positions and on induction of hypercapnia. Results are expressed as mean ± standard error of mean.

Pulse amplitude (PA) was lower in the glaucoma group in all positions, significantly so when supine (*P* = 0.036) (Figure 3(a)). PA reduced in the supine position in NTG by 13.9% (*P* = 0.02) and a more minor reduction of 7.4% was seen in the control group (*P* = 0.61). Hypercapnia induced a significant increase in PA in NTG of 14.76% (*P* = 0.004) that was not seen in the control group (*P* = 0.37). Hypercapnic PA was marginally less in the glaucoma group (mean PA NTG = 2.41 mmHg; control = 2.45 mmHg).

#### 3.4.2. Pulse Volume

Pulse volume (PV) was lower in the glaucoma group at all points during measurements (Figure 3(b)). Sitting and supine values were significantly lower compared with controls (sitting *P* = 0.03; supine *P* = 0.009). NTG patients experienced a significant drop in PV of 17.6% once supine (*P* = 0.005), whereas controls showed little change with a mean increase of 1.6% (*P* = 0.7). Hypercapnia induced a significant change in the glaucoma group of 24.2% (*P* = 0.001) but little increase in controls (3.01%, *P* = 0.87). PV values in hypercapnia were closer for the 2 groups but still higher in controls.

#### 3.4.3. Pulsatile Ocular Blood Flow

**Figure 4:**
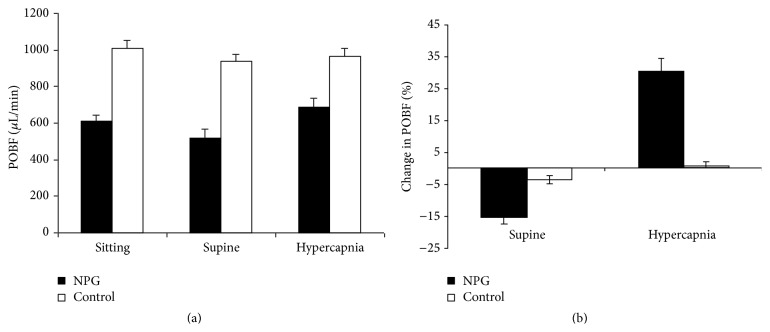
Pulsatile ocular blood flow. Pulsatile ocular blood flow (POBF) in microlitres per minute (*μ*L/min) and percentage (%) change in POBF in normal tension glaucoma patients (NTG) and healthy volunteers (control) in sitting and supine positions and on induction of hypercapnia. Results are expressed in mean ± standard error of mean.

Pulsatile ocular blood flow (POBF) was significantly lower in NTG than controls in all 3 measurement situations (sitting and supine *P* = 0.00001; hypercapnia *P* = 0.008) (Figure 4(a)). Supine position significantly reduced POBF in glaucoma patients with a drop of 15.19% (*P* = 0.03) but had minimal effect on controls that showed a mean drop of 3.4% (*P* = 0.3) (Figure 4(b)). Hypercapnia induced a highly significant increase in POBF values in NTG with a mean increase of 30.4% (*P* = 0.0001) but little change was noted in controls whose mean change was 1.1% (*P* = 0.7). The difference between the 2 groups was greatly reduced with hypercapnia but remained significant (*P* = 0.008) (Figure 4).

#### 3.4.4. High and Low Responders

**Figure 5:**
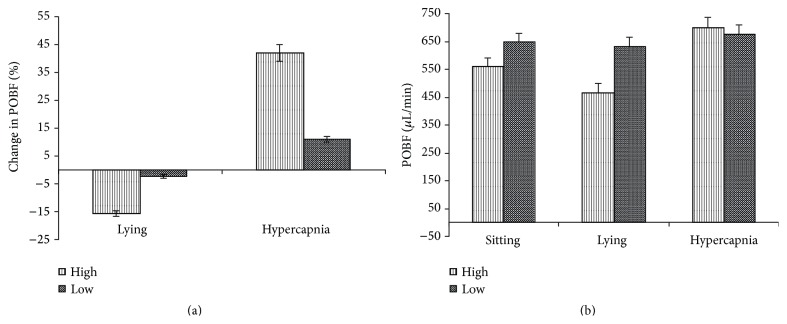
Pulsatile ocular blood flow in hypercapnic responders. Pulsatile ocular blood flow (POBF) in microlitres per minute (*μ*L/min) and percentage (%) change in POBF in high (High) and low (Low) hypercapnic responders in sitting and supine positions and on induction of hypercapnia. Results are expressed in mean ± standard error of mean.

Within the glaucoma group, there was a clear division found on the basis of hypercapnic response. 13 patients were deemed to be high responders, having shown an increase in POBF of 20% or more in response to hypercapnia, and 7 were low responders showing less than 20% of an increase (Figure 5(a)).

Demographic data of these 2 groups showed no significant difference in age (*P* = 0.89), sex distribution (*P* = 0.6), IOP (Goldmann) (*P* = 0.13), axial length (*P* = 0.4), or visual field indices (md *P* = 0.35; cpsd *P* = 0.66) between the two groups. Medical history did reveal some differences; 6 of the high responders gave a history of borderline hypertension compared with 2 of the low responders (*P* = 0.095). Seven high and 4 low responders gave a history of migraine and/or Raynaud's type circulation (*P* = 0.75) and 2 high and 3 low responders had a history of a severe hypovolemic or hypotensive episode in the past (*P* = 0.15).

MAP and HR were similar in the two groups and did not differ significantly during postural change or on induction of hypercapnia. Systolic BP increased in both subgroups with hypercapnia but there was no difference between the two in the magnitude of increase.

The high responders tended to have a lower POBF, significantly so in the lying position (*P* = 0.008) (Figures 5(a) and 5(b)). This group also exhibited a more pronounced reduction in POBF (*P* = 0.02) on lying down with a mean reduction of 17.4% as compared with a low responder drop of 1.4% (Figure 5(b)). The mean increase with hypercapnia was 41.18% in the high responders and 8.93% in the low (*P* = 0.00001).

### 3.5. Digital Blood Flow Levels

**Figure 6:**
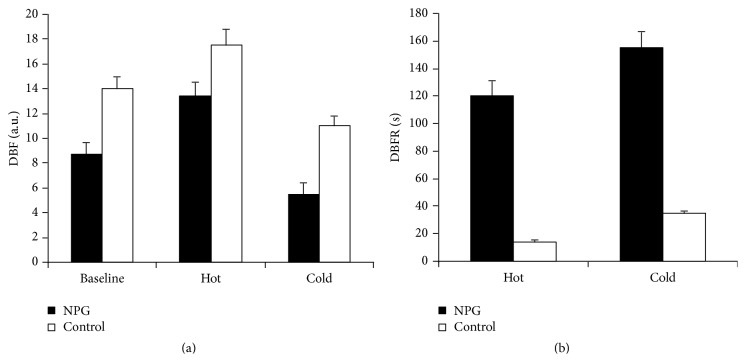
Digital blood flow and recovery times. Digital blood flow (DBF) in arbitrary units (a.u.) and digital blood flow recovery times (DBFR) in seconds (sec) in normal tension glaucoma patients (NTG) and healthy volunteers (control) at baseline and following hot and cold immersion. Results are expressed as mean ± standard error of mean.

Mean baseline flow was significantly lower in the glaucoma group (8.7 a.u.) than in controls (14.1 a.u.) (*P* = 0.02). Following hot immersion, the glaucoma group showed a greatly increased flow (53.68%; *P* < 0.001) whilst controls showed a moderate, nonsignificant increase (24.11%; *P* = 0.09). Cold immersion resulted in a significant drop in DBF in NTG subjects (42.52%; *P* = 0.003). Again controls showed a nonsignificant change (21.98%; *P* = 0.1) (Figure 6(a)).

### 3.6. Digital Blood Flow Recovery Times

DBF recovery times were also altered in NTG (Figure 6(b)). Following heat immersion, NTG subjects took a mean of 120 seconds to return to baseline flow compared with a control recovery time of 14 seconds. The difference between the 2 groups was significant (*P* = 0.03). Recovery from cold immersion was even more prolonged in the glaucoma group with a mean recovery time of 155 seconds compared with a control recovery of 35 seconds (*P* = 0.002).

### 3.7. High and Low Hypercapnic Responders

**Figure 7:**
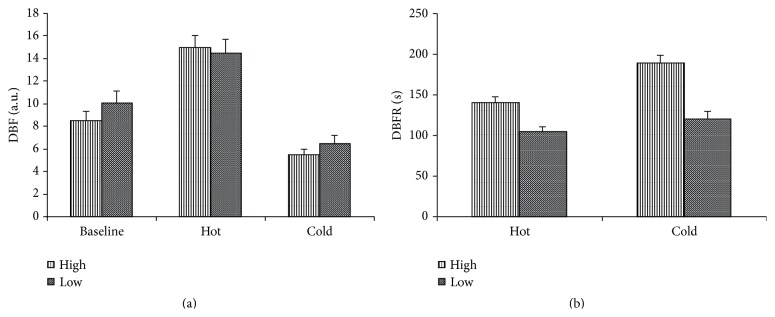
Digital blood flow and recovery times in hypercapnic responders. Digital blood flow (DBF) in arbitrary units (a.u.) and digital blood flow recovery times (DBFR) in seconds (secs) in high (High) and low (Low) hypercapnic responders at baseline and following hot and cold immersion. Results are expressed as mean ± standard error of mean.

**Figure 8:**
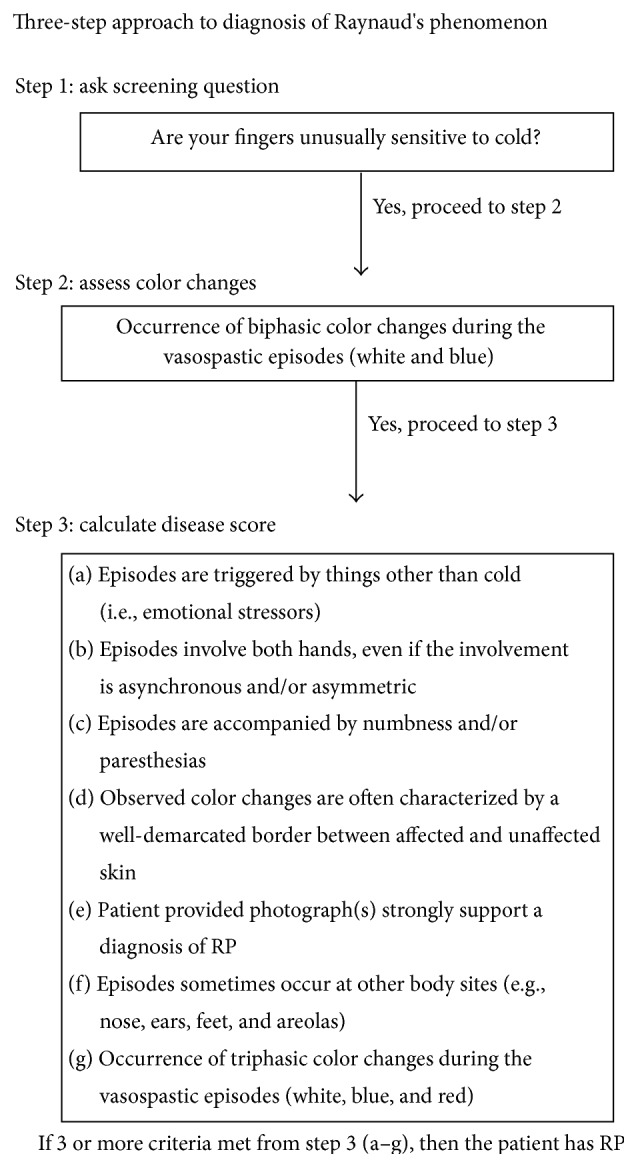


The glaucoma group had been divided into 2 subgroups based on the level of their POBF response to hypercapnia (Figures 7(a) and 7(b)). Baseline blood flow levels were lower in the high response group (mean high DBF: 8.5 a.u.; mean low DBF: 10.1; *P* = 0.07). Flow increased significantly in both groups following heat immersion but much more so in the high response group who showed a mean % change of 76.4% (*P* < 0.0001) compared with a low responder change of 43.5% (*P* = 0.03).

Cold immersion reduced flow in both subgroups significantly but again the degree of reduction was greater in the high responders with a mean % change of 41.17% compared to the low responder change of 32.98% (*P* = 0.04 in high responders and *P* = 0.07 in low responders).

Blood flow recovery from heat immersion was prolonged in both groups with a mean of 141.8 seconds in the high response group and a low response mean of 102.3 seconds. Cold recovery was significantly more prolonged in the high responders: mean 192 seconds compared to low responders with a mean of 114.4 seconds (*P* = 0.03).

## 4. Discussion

This study has shown that POBF was significantly lower in patients with untreated NTG. Within the glaucoma group, there were 2 distinct subgroups based on the magnitude of POBF response to hypercapnia.

POBF was lower at baseline, perhaps suggesting a resting vascular tone, which favours constriction. Supine position caused a significant drop in POBF in glaucoma patients, further suggesting a failure of the autonomic system to regulate ocular blood supply adequately. Hypercapnia induced an exaggerated ocular vasodilation in the glaucoma group, which may reflect relative vasoconstriction in the resting tone and also the possible reversibility of this.

Consistent with previous research, no significant differences in the average diurnal and nocturnal BP variables were found between the NTG and control groups [61, 62]. The relationship between systemic BP and glaucoma is controversial. A number of studies have shown an association between hypotension and nocturnal BP dipping and glaucoma [63–65]. However this finding has been contradicted in another work [61, 66]. In keeping with the findings of the Rotterdam Study [67], we found that diastolic BP was elevated compared to normal controls. In the Rotterdam eye study, patients with an ocular perfusion pressure lower than 50 mmHg had a four-times greater risk of developing OAG than those with a perfusion pressure of 80 mmHg [67].

The postural decrease in BP and HR shown by healthy volunteers both in this study and others is a feature of autonomic integrity [68, 69]. During postural change from upright to supine, gravity decreases and therefore a lower systemic BP is required to ensure adequate blood supply to all organs and tissues, achieved by reducing peripheral vascular resistance. This has a knock-on effect of reducing cardiac output resulting in a lower HR. Organs and tissues, which have intact autoregulation, are able to maintain blood flow during postural change. The remainder of the body relies on either the autonomic system or local regulatory mechanisms. An abnormal change in haemodynamic parameters during postural change may be an indication of vascular irregularity. This research suggests that a lack of significant change in both diastolic BP and HR may reflect a dysfunction in the normal mechanisms controlling this response, namely, the autonomic system [70].

It appears from baseline measurements that NTG patients have relatively constricted vessels in the ocular circulation, but the exaggerated response to hypercapnia suggests that this reduction in baseline flow is reversible and that the ocular circulation is responsive to vasodilatory stimulation. The absence of such a response in healthy volunteers may indicate that they control their vascular responses in a more efficient way. Alternatively they may simply have less potential for vasodilation as their vessels do not demonstrate the degree of vasoconstriction seen in their NTG counterparts.

Our work corroborates other studies that demonstrate that hypercapnia causes an increase in ocular blood flow [44, 49]. In the cerebral and ocular circulations, which are primarily autoregulated, hypercapnia-induced vasodilation is mediated by nitric oxide (NO) [71, 72]. This role for NO would certainly fit with the theory that the baseline impairment of POBF is due to an imbalance in local constricting and dilating factors. If this is the case, then the release of endothelium mediated NO by hypercapnia may, at least partially, correct this imbalance.

DBF was basally reduced, overly responsive to temperature changes, and demonstrated prolonged recovery from such provocation. These features were widespread in the glaucoma group studied even though just over half of them reported vasospastic symptoms.

A subgroup of the glaucoma patients had a particularly high hypercapnic response, and these subjects had significantly lower baseline POBF and showed a greater drop in PODF in the supine position. They also exhibited a greater degree of digital vasospasm with greater cold response and longer cold recovery times. These findings closely correlate with studies from other groups [9]. This subset appeared to have an underlying primary vascular dysregulation (PVD). PVD has been previously described by Flammer et al. to be the underlying pathogenic process in NTG [10, 73]. The ability to treat this underlying PVD will likely provide the basis of future treatments for NTG [74, 75].

Calcium channel blockers (CCBs) cause an inhibition of calcium influx into cells, leading to relaxation of vascular smooth muscle and consequently increased blood flow in various organs [76, 77]. Several investigators have reported in experimental conditions that CCBs relax isolated retinal or ciliary arteries [78–82]. Nilvadipine caused a 30–40% increase in the ONH and choroidal blood velocity with little systemic effects [83]. Recently, Nilvadipine, a dihydropyridine (DHP) calcium channel antagonist, has become the focus of a large multicentered randomised control trial investigating the potential neuroprotective properties of the drug in Alzheimer's disease [84, 85]. Intravenous lomerizine has been shown to both increase the ONH blood flow and inhibit endothelin-1 induced hypoperfusion of the ONH [86–89]. One of the main therapeutic properties attributed to CCB is their ability to increase cerebral blood flow [90]. The question as to whether a similar neuroprotective advantage could be achieved in the NTG “high responder” group of patients is worthy of further investigation.

The combined findings of these indirect assessments of vascular function would suggest a disorder of the usual control mechanisms governing ocular and DBF and also systemic haemodynamics. Both the autonomic system and local ocular regulatory mechanisms, namely, the vascular endothelium, are implicated by these findings.

## Figures and Tables

**Table 1:** Demographic comparison of patients with normal tension glaucoma (NTG) and healthy volunteers (control).

	NTG (*n* = 20)	Controls (*n* = 18)	*P* value
Age (years)	58.72 ± 10.0	57.5 ± 9.6	0.71
Sex (M : F)	11 : 9	9 : 9	
Ax. L (mm)	24.03 ± 1.22	23.6 ± 0.89	0.27
IOP (mmHg)	16.6 ± 2.97	14.31 ± 3.2	0.12
MAP (mmHg)	103.45 ± 13.53	95.96 ± 12.88	0.11
HR (b/min)	66.7 ± 13.06	72.75 ± 14.59	0.22
CVS disease	8	3	0.092
Hypovol/Hypoten	5	0	**0.018**
Vasospasm	11	3	**0.008**

Results are mean ± standard error mean and were compared using *t*-test. Male to female ratio (M : F); intraocular pressure (IOP) in millimetres of Mercury (mmHg); axial length (Ax. L) in millimetres (mm); mean arterial blood pressure (MAP); heart rate (HR) in beats per minute (b/min); cardiovascular disease (CVS disease); history of hypovolaemia or hypotension (Hypovol/Hypoten).

Bold Signifies statistically significant *P* < 0.05.

## Appendix

### International Consensus Criteria for the Diagnosis of Raynaud's Phenomenon

See Figure 8.
